# The future of physiological research: A greater understanding of female master athletes and aging?

**DOI:** 10.14814/phy2.70109

**Published:** 2024-10-31

**Authors:** Lorcan S. Daly

**Affiliations:** ^1^ Department of Sport and Health Sciences Technological University of the Shannon Athlone Ireland; ^2^ SHE Research Centre, Technological University of the Shannon Athlone Ireland

**Keywords:** age‐related performance decline, aging physiology, gender gap, healthspan, menopause

## Abstract

High caliber master athletes provide a valuable model for studying inherent physiological aging and performance capacity, without the confounding factor of physical inactivity. Despite the remarkable achievements of female master athletes, their participation rates remain significantly lower than those of their male counterparts, particularly at more advanced ages. This review examines the biological sex gap in sports participation among master athletes and the subsequent disparity in empirical research, thereafter exploring possible contributing factors. It highlights the importance of studying female master athletes to better understand the aging process and offers recommendations to address current evidence gaps. The need for more comprehensive mechanistic data on highly trained older women, novel cataloguing and analysis of real‐world datasets, case studies/series, and longitudinal research are also emphasized. Although analyzing the records of female master athletes as a surrogate to determine age‐related physiological and performance changes is a common approach, the process may be hindered by the considerably lower participation rates of women. Therefore, an important step toward bridging these gaps is the longitudinal, integrative study of female athletes engaged in lifelong exercise. Such analyses would improve our understanding of senescence in women and may inform interventions targeting the promotion of physical function in older adults.

## Graphical abstract

High calibre master athletes provide a valuable model for studying inherent physiological ageing and performance capacity. Female master athletes participation rates, and their subsequent representation in empirical research, remain significantly lower than males, particularly at advanced ages. This review emphasises the need for more comprehensive mechanistic data on highly‐trained older women, analysis of large real‐world datasets, case studies/series, and longitudinal research to improve understanding of female senescence.

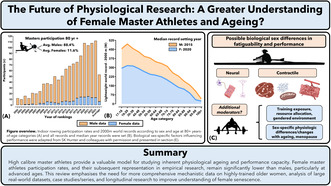

## 1 INTRODUCTION

Remarkable sporting performances at advanced ages are widely demonstrated by master athletes, for example, Jeanne Rice running a marathon in 3:33:27 at 75 years of age (World Athletics Outdoor Roadrunning Marathon Records, 2024). Such feats of athleticism are indicative of highly functioning cardiovascular, respiratory, neuromuscular, and metabolic systems synergistically working in concert (Daly et al., 2023; Lazarus & Harridge, 2017; Tanaka & Seals, 2008). Given the deterioration of these systems typically observed during aging, exploring avenues to promote longevity and physical/cognitive function in older individuals bears significant importance (Lazarus & Harridge, 2017; Roman et al., 2016; Tanaka & Seals, 2008). Subsequently, examining the physiology of master athletes, and the training and nutritional practices which undergird their remarkable functional characteristics, can present valuable insights (Daly et al., 2023; Tanaka & Seals, 2008).

The use of master athletes to represent an exemplary rate of aging‐dependent physiological decline is common practice in empirical literature (Ganse et al., 2021; Hoog Antink, Braczynski, Kleerekoper, et al., 2021; Hunter, 2024; Lazarus & Harridge, 2017). These athletes, who continue to engage in high‐intensity training regimens into advanced ages, provide a unique opportunity to study the impacts of aging while controlling for the confounding effects of physical inactivity (Lazarus & Harridge, 2017; Tanaka & Seals, 2008). Nevertheless, examining master athletes for such purposes is not without limitations (Hunter, 2024). Foremost, a significant gender gap exists in master participation rates, likely underpinned in principle by sociocultural factors (Hunter & Stevens, 2013), resulting in substantially less female data (Ganse et al., 2020; Hunter, 2024). Therefore, this report will briefly (i) summarize relevant sex‐specific experimental, case study/series, and longitudinal findings, and (ii) present novel applied data in rowing as a sporting example. Hereafter, the future landscape of aging physiological research, with a particular focus on female master athletes as a vehicle to contribute important evidence, will be inferred upon.

## 2 GENDER DIFFERENCES IN ATHLETIC PARTICIPATION AND MASTERS RESEARCH

**FIGURE 1:**
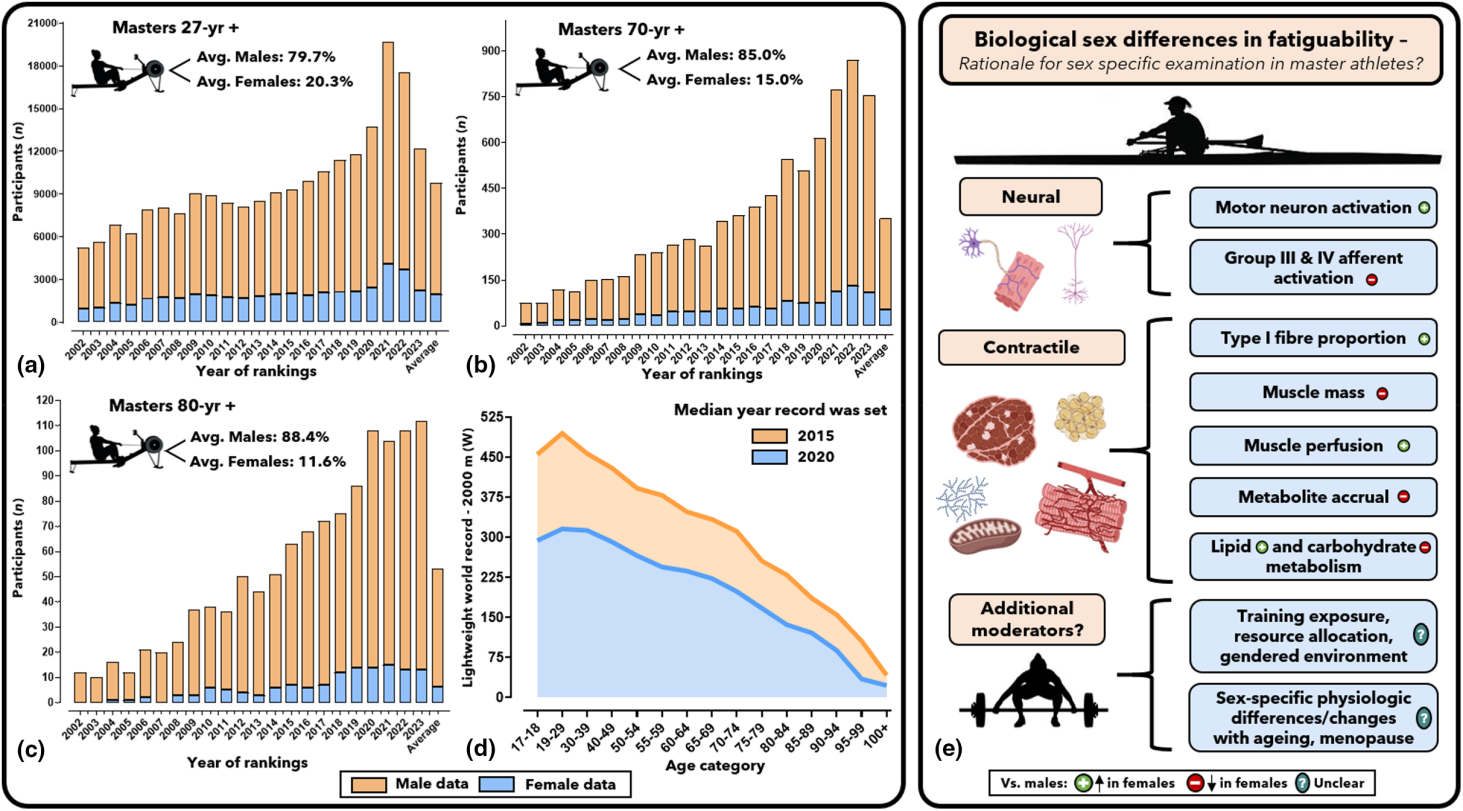
Indoor rowing participation rates according to sex for (a) 27+, (b) 70+, and (c) 80+ years‐of‐age categories. Additionally, (d) shows the lightweight indoor rowing 2000 m world records by sex, presented as mean power output (W), along with the median year each record was set. Finally, (e) outlines biological sex‐specific factors possibly influencing performance, adapted from Hunter (Hunter, 2024), with permission. The indoor rowing data were sourced from Concept2 (as of May 20, 2024) and processed; full data are available at an open‐source repository: https://rb.gy/ir3wcF.

Analyses of various athletic records have suggested that performances decline exponentially after approximately the seventh/eighth decade of life, with a more pronounced senescence in women when compared to men (Hunter, 2024; Lazarus & Harridge, 2017; Tanaka & Seals, 2008). This observation has led to the hypothesis that women may experience more severe physiological decrements during aging (Lazarus & Harridge, 2017; Tanaka & Seals, 2008), with putative mechanisms possibly relating to menopause (e.g., deteriorated vascular responsiveness) (Bondarev et al., 2021; Hulteen et al., 2023; Tamariz‐Ellemann et al., 2023; Tanaka & Seals, 2008). Nevertheless, several potential biases require consideration (Hunter, 2024; Hunter & Stevens, 2013). Foremost, sociocultural, legal, and other barriers have historically limited women's sports participation (Hunter, 2024; Hunter & Stevens, 2013). For instance, the 1928 Olympics held the first women's 800 m race and reports falsely claimed that numerous women dropped out or collapsed from exhaustion (DeFrantz, 1997). Despite no such incidents occurring, these reports led to the event's removal for 32 years (DeFrantz, 1997). The gendered environment of sport and exercise, shaped by institutions, media, policies, and cultural/historical norms (e.g., female sport participation historically framed as “dangerous” owing to “unfit biology”) (Parsons et al., 2021), often discourages girls and women from sports participation, and limits their access to resources and opportunities (Hunter, 2024; Hunter & Stevens, 2013). Such barriers may help explain the significantly lower rates of female sports participation, particularly among older female masters, who likely experienced a more pronounced gendered environment in earlier decades (DeFrantz, 1997; Parsons et al., 2021). Accordingly, reduced female master participation rates could have conceivably lowered performance records, thereby precluding an accurate overview of female aging through this lens (Hunter, 2024; Hunter & Stevens, 2013). Indeed, earlier work (Hunter & Stevens, 2013) has reported that increased participation among older adults was associated with improved performances, particularly for women. Despite improving trajectories, female participation remains considerably lower than males, and this discrepancy widens with higher ages in running (Hunter & Stevens, 2013), swimming (Hunter, 2024), throwing events, (Ganse et al., 2020) and other sports (Hunter, 2024). Serving as a novel example, Figure 1 outlines indoor rowing masters participation rates, 2000 m world record trajectories and possible sex‐specific considerations influencing master records (Hunter, 2024).

Data from Figure 1 suggest female participation is increasing, yet remains substantially lower than males, with the gap accentuated in older categories. Subsequently, current female masters records may potentially be constrained by participation (Ganse et al., 2020; Hunter & Stevens, 2013), while future increases in participation could conceivably result in elevated records and less pronounced age‐related decline (Hunter & Stevens, 2013). This premise is bolstered by the more recent emergence of female master records when compared with males (median year: 2020 vs. 2015: Figure 1d). Additionally, the possibility that the most gifted athletes retire before ever competing in master events could exaggerate age‐related record declines (Lazarus & Harridge, 2017). Therefore, longitudinally tracking athletes competing across their lifespan, rather than single athletic records/events, is needed to offer more reliable insights (Ganse et al., 2020; Hoog Antink, Braczynski, Kleerekoper, et al., 2021).

## 3 THE ROLE OF LONGITUDINAL AND/OR CASE STUDIES

Longitudinal studies are vital for understanding human senescence, as they can track physiological changes and overcome potential biases of record analysis (Ganse et al., 2021; Hoog Antink, Braczynski, Kleerekoper, et al., 2021). Indeed, it has been suggested that the aging‐associated declines are less severe when looking at longitudinal performances, rather than single results/records (Ganse et al., 2020; Hoog Antink, Braczynski, Kleerekoper, et al., 2021). Notably, the Swedish veteran athletics database, the largest longitudinal dataset on master performances available to date, has been widely analyzed (Ganse et al., 2020, 2021; Hoog Antink, Braczynski, & Ganse, 2021). Despite its extensive scale, studies have elected not to analyze the female athletes given their far lesser representation (Ganse et al., 2020; Hoog Antink, Braczynski, Kleerekoper, et al., 2021), underscoring the need for further high‐quality evidence in the population. At present, although case analyses are poorly documented, female master athletes have demonstrated notable physiological characteristics, such as an 83‐year‐old runner with a 42.3 mL kg min^−1^ *V̇*O_2max_ (Cattagni et al., 2020), or a 71‐year‐old powerlifter with a well‐developed Type II muscle fiber cross‐sectional area (4536 μm; +46% vs. age‐matched controls) and bone mineral density (1.09 g cm^2^) (Fuchs et al., 2024). Noteworthy, despite aggregated data precluding sex‐specific examination, recent research has demonstrated significant muscle hypertrophy and strength gains in advanced‐aged individuals, implying robust adaptive capacities in both males and females >85‐years of age (Marzuca‐Nassr et al., 2023).

Notably, between‐sex variability in contractile and neural physiology (e.g., Figure 1), such as muscle fiber typology, glycolytic/fat metabolism, sarcoplasmic reticulum Ca^2+^ATPase activity, mitochondrial protein homeostasis and density/function, PaO_2_ and motor neuron activation (Hunter, 2024; Hunter & Stevens, 2013; Roman et al., 2016), predicate the need for sex‐specific investigations. Indeed, estrogen, which may elicit protective effects on the functional capacity of the skeletal, cardiovascular and neuromuscular systems, is significantly lowered during menopause, possibly impairing older females' physiological reserves (e.g., vasodilator responsiveness, isometric/dynamic strength, bone mineral density, and body composition) (Bondarev et al., 2021; Hulteen et al., 2023; Roman et al., 2016; Tamariz‐Ellemann et al., 2023). Consequently, such changes may plausibly implicate performance capacity decrements (Hulteen et al., 2023; Tamariz‐Ellemann et al., 2023). Therefore, long‐term tracking is a necessary prerequisite to robustly profile aging females' physiologic reserves/performance trajectories. As highlighted by Joyner and colleagues (Joyner et al., 2011), “experiments of nature”—such as studying McCardle's patients to better understand the ventilatory threshold, or comparing bus drivers and conductors to examine the role of physical activity in health—can help close important knowledge gaps. Similarly, future case series monitoring female master athletes, ideally integrative and well‐sampled, can deliver insight toward the lifelong effects of intensive exercise training (Hunter, 2024; Joyner et al., 2011).

## 4 ON THE HORIZON: FURTHER ANALYSES IN FEMALES ACROSS THE LIFESPAN

Future collective approaches involving mechanistic and interventional research, “big data” analyses and longitudinal studies of female master athletes hold promise to enhance the current literature base. Below are areas that may yield key contributions:

1. *Mechanistic and interventional work*—Further high‐quality evidence is necessary to elucidate sex‐specific trajectories of physiological functional capacities during aging when (i) training in youth and detraining, (ii) lifelong training and (iii) commencing training at advanced ages. Comprehensive integrative physiological assessments involving morphological, neuromuscular, cardiorespiratory, vascular, and hematopoietic measures should accompany lifestyle monitoring (e.g., nutritional intake, training practices, and psychological motivation) to provide global mechanistic insights into the factors underlying performance changes. Furthermore, research avenues exploring sex‐specific outcomes for older athletes' (i) post‐exercise recovery kinetics, (ii) nutritional strategy efficacy, (iii) sleep requirements, (iv) injury prevention/rehabilitation, and (v) post‐exercise adaptive signaling should be examined.
2. *Analysis of* “*big data*”—Growing female master participation rates and the increasing availability of expansive, digitally recorded datasets from athletic events/training (e.g., Zwift, Strava or Concept2 records) present unique opportunities for highly powered longitudinal analyses and the provision of important insights. Analytical methods such as multilayer machine learning approaches, which have outperformed quadratic/linear regression models in similar datasets (Hoog Antink, Braczynski, & Ganse, 2021), may reveal important patterns and trends undetectable with smaller samples. Moreover, big data can be useful to determine age‐specific percentile benchmarks for performance, critical power, and power‐law computations.
3. *Case series*—Establishing a robust evidence base in highly trained female master athletes' performance trajectories in both neuromuscular‐ and endurance‐emphasized sports would provide valuable insight and elicit important research and practical applications. With increasing participation, resources and empirical interest in female master athletes, significant research progress is anticipated.

## 5 CONCLUSION

This report underscores the critical role of studying female master athletes in understanding inherent aging processes and offers suggestions to address existing evidence gaps. It emphasizes the need for more comprehensive mechanistic data in highly trained older women, further cataloguing of real‐world databases, case reports/series, and longitudinal interventions. Notably, analyzing female master athletes' records across the lifespan to profile age‐related physiological and performance changes is a relatively common approach but may be undermined by substantially lower female participation rates compared to males. Subsequently, an important step to bridge these data gaps is the integrative, longitudinal study of female senescence in those engaged in lifelong exercise training. As proposed by Hunter (Hunter, 2024), future work assimilating well‐controlled laboratory findings with real‐world data may lead to a transformative understanding of female aging, consequently laying the groundwork for improved health guidelines and interventions.

## FUNDING INFORMATION

This review article did not receive any supporting funding.

## CONFLICT OF INTEREST STATEMENT

The author has no conflict of interest to declare, financial or otherwise.

## ETHICS STATEMENT

This study did not require institutional ethical approval as it involved a review of the literature and analysis of publicly available, open‐access data. No primary data collection involving human or animal subjects was conducted.

## Data Availability

The data supporting Figure 1 of this study, including World Rowing Participation rates via ranking world records computed as mean power (Watts), are openly available at the following open‐access data repository for verification or future use: https://rb.gy/ir3wcf. For further questions or additional information, please contact the author.
